# Non-Coding RNAs are Differentially Expressed by *Nocardia brasiliensis in Vitro* and in Experimental Actinomycetoma

**DOI:** 10.2174/1874285801711010112

**Published:** 2017-06-30

**Authors:** Josué S. Cruz-Rabadán, Juan Miranda-Ríos, Guadalupe Espín-Ocampo, Luis J. Méndez-Tovar, Héctor Rubén Maya-Pineda, Francisca Hernández-Hernández

**Affiliations:** 1 Departamento de Microbiología y Parasitología, Facultad de Medicina, Universidad Nacional Autónoma de México (UNAM), Ciudad de México, México.; 2 Unidad de Genética de la Nutrición, Instituto Nacional de Pediatría and Departamento de Biología Molecular y Biotecnología, Instituto de Investigaciones Biomédicas, UNAM, Ciudad de México, México.; 3 Departamento de Microbiología Molecular, Instituto de Biotecnología, UNAM, Cuernavaca, Morelos, México.; 4 Hospital de Especialidades, Centro Médico Nacional Siglo XXI, Instituto Mexicano del Seguro Social, Ciudad de México, México.

**Keywords:** *Nocardia brasiliensis*, Non-coding RNAs, Small RNA, Actinomycetoma, RNA-Seq, Actinomycetes, Mycetoma

## Abstract

**Introduction:**

*Nocardia* spp. are common soil-inhabiting bacteria that frequently infect humans through traumatic injuries or inhalation routes and cause infections, such as actinomycetoma and nocardiosis, respectively. *Nocardia brasiliensis* is the main aetiological agent of actinomycetoma in various countries. Many bacterial non-coding RNAs are regulators of genes associated with virulence factors.

**Objective:**

The aim of this work was to identify non-coding RNAs (ncRNAs) expressed during infection conditions and in free-living form (*in vitro*) in *Nocardia brasiliensis*.

**Methods and Result:**

The *N. brasiliensis* transcriptome (predominately < 200 nucleotides) was determined by RNA next-generation sequencing in both conditions. A total of seventy ncRNAs were identified in both conditions. Among these ncRNAs, 18 were differentially expressed, 12 were located within intergenic regions, and 2 were encoded as antisense of 2 different genes. Finally, 10 of these ncRNAs were studied by rapid amplification of cDNA ends and/or quantitative reverse transcription polymerase chain reaction. Interestingly, 3 transcripts corresponded to tRNA-derived fragments (tRNAs^Cys, Met, Thr^), and one transcript was overlapped between an intergenic region and the 5´end of the 23S rRNA. Expression of these last four transcripts was increased during *N. brasiliensis* infection compared with the *in vitro* conditions.

**Conclusion:**

The results of this work suggest a possible role for these transcripts in the regulation of virulence genes in actinomycetoma pathogenesis.

## INTRODUCTION

Bacteria inhabit a broad spectrum of ecological niches and are subject to a great diversity of environmental conditions. Bacteria mainly consist of saprophytic and commensal species; however, bacteria also comprise pathogenic species that colonize and successfully confront the host’s immune system. Therefore, pathogenic bacteria require strict control of virulence gene expression and stress-response mechanisms. Until recently, this control was attributed to the activity of transcription factors that controlled the activity of sets of genes in response to environmental conditions. Recently, attention has been focused on the participation of non-coding RNAs (ncRNAs, also called small RNAs (sRNAs)) in the regulation of bacterial pathogenesis [1]. In many bacteria, most RNA regulators range in size from 50 to 300 nucleotides (nt) and have a partial complementarity to their target mRNAs [2, 3].

The most extensively studied ncRNAs in bacteria are those called *trans*-encoded sRNAs, and they regulate mRNAs by short, imperfect base-pairing interactions. The antisense RNAs (asRNAs) act in *cis* and are encoded within the complementary strand to regulate gene expression [4]. Other regulatory molecules include CsrB RNA, which binds to the protein CsrA, counteracting its translational repressor activity [2]. Recently, a class of tRNA-derived ncRNAs has been described in different eukaryotic organisms [5]. In addition, in the halophilic archaeon *Haloferax volcanii* grown under different temperature, growth phases and salt concentration conditions, Heyer *et al.* found 145 ncRNAs in intergenic regions (IGRs) and 45 anti-sense by RNA sequencing (RNA-Seq). This study revealed an important number of tRNA-derived fragments corresponding to different tested conditions [6]. In another study with *H. volcanii,* Gebetsberger showed that tRNA is processed in a stress-dependent manner. A tRNA-derived fragment (Val-tRF) binds to the small ribosome subunit in *in vitro* and *in vivo* conditions. As a consequence, Val-tRF reduces protein synthesis by interfering with peptidyl transferase activity [7].

*Nocardia brasiliensis* is a soil-inhabiting filamentous Gram-positive bacterium with a 9.4-Mb chromosome genome that exhibits a high G-C content (68%) and encodes 8,414 predicted proteins. Several open reading frames (ORFs) coding for virulence factors, such as catalases, superoxide dismutases, phospholipase C, haemolysins and proteases have been identified [8]. This actinomycete mainly causes two clinical forms of infection: nocardiosis and actinomycetoma. Actinomycetoma is a chronic disease mainly observed in males from rural areas and results from the traumatic implantation of actinomycetes from the soil into the tissues. The lesions consist of suppurating abscesses, granulomata and draining sinuses with the presence of “grains”, which are compact micro-colonies of the aetiological agent [9]. In the first hours and days after the *Nocardia* inoculation in experimental actinomycetoma, an inflammatory infiltrate, consisting mainly of macrophages and neutrophils, is observed [10]. By the fiftieth day, typical bacterial granules are present. After six months of evolution, numerous granulomatous foci formed by neutrophils, macrophages, foam cells and fibrotic tissue are observed. Therefore, *N. brasiliensis* adapts well to host´ immune system by inducing a vicious cycle of inflammation followed by tissue damage and subsequent inflammation. It has been suggested that this response involves molecular mediators, such as the Toll-like receptors (TLR2 and TLR4), that may form the basis of chronic inflammation [10, 11]. In Mexico, 65.58% of actinomycetoma cases are caused by *N. brasiliensis* [12].

The presence of ncRNAs has not been explored in *N. brasiliensis*. The aim of this study was to identify the ncRNAs in *N. brasiliensis* during murine experimental actinomycetoma and in *in vitro* conditions using RNA-Seq and bioinformatic tools.

## MATERIALS AND METHODS

### Bacterial Strain and Culture Conditions

The strain *N. brasiliensis* HUJEG-1 (ATCC700358) was used. Bacteria were grown on brain heart infusion (BHI) agar (BD-bioxon, cat. no. 210700) for eight days at 28°C. A suspension equivalent to 3X10^9^ colony-forming units (C.F.U.)/mL was prepared as an inoculum. Two strains of *Escherichia coli* (XL1-BLUE and Stellar^TM^) were used as competent cells for cloning experiments.

### Induction of Murine Actinomycetoma

Ten male CD-1 mice (25 g and eight weeks of age) were used in experiments. Animals were managed according to the Research and Ethics Committee Code of the Faculty of Medicine, UNAM (Project 011-2012). We followed the 8^th^ Edition of Guide for the Care and Use of Laboratory Animals when caring for and using the mice in our study [13]. To induce *N. brasiliensis* infection, 50 µl of the previously prepared suspension were inoculated in the murine plantar pad. After bacterial inoculation, mice were maintained for two months at room temperature (between 24 and 28°C) and were provided food and water *ad libitum*. After this period, the murine actinomycetoma was clinically evident (by swelling and distortion of the foot, sinus tracts and drainage of purulent exudate), and mice were sacrificed by cervical dislocation [13]. The plantar pads were washed, and under sterile conditions, purulent exudate was completely extracted and deposited into sterile 1.5 mL tubes on ice. This material was washed three times with sterile saline solution and recuperated by centrifugation.

### RNA Next-Generation Sequencing

For total RNA extraction, one hundred milligrams of purulent exudate (formed mainly by bacterial micro-colonies, i.e., grains) were macerated in the presence of liquid nitrogen until obtaining a fine, whitish dust. To purify RNA that is highly enriched for small RNAs species (mainly 200 nt or less), the mirVana^TM^ PARIS^TM^ Kit (Life Technologies Corporation, cat. no. AM1556) was used following the manufacturer´s instructions. The quality of this RNA preparation was verified in a denaturing acrylamide gel (8 M urea (Sigma, cat. no. U1250) and 12% acrylamide (Bio-Rad, cat. no. 161-0146)). Five hundred nanograms of purified RNA were used to synthesize cDNA and subjected to next-generation sequencing in duplicate (LCSciences, Texas, USA) using an Illumina HiSeq 2500 platform and samples were run in the 50 cycle SE read configuration for 36 bp single-end reads. In this system the adapters (5´ and 3´) are ligated to the RNA and after an RT reaction is used to create single stranded cDNA which is then PCR amplified using the TruSeq Small RNA Sample Prep kits. Sequences were mapped against reference databases, such as miRBase, to separate sequences corresponding to mouse transcripts. Non-mapped sequences in miRBase were grouped as no-hits for mouse and were mapped against the *N. brasiliensis* HUJEG-1 genome. As a control, an *N. brasiliensis* culture grown on BHI agar and incubated at 28°C for eight days was used. This culture was also processed to obtain and sequence the small RNAs using the same conditions as mentioned above including 500 ng of sRNA as starting material.

### Rapid Amplification of cDNA Ends (RACE)

RACE is a PCR-based method used to obtain the full-length sequence of the 5´ and 3´ ends of a transcript and therefore its size [14]. For this procedure, cDNA was obtained from purified ncRNAs using random primers following the manufacturer’s instructions (Clontech, cat. no. 634859). The transcripts of the 5’ and 3’ ends were amplified using the corresponding specific oligonucleotides (Table S2). The amplicons were cloned using the Thermo Scientific CloneJET PCR Cloning kit (Fermentas, cat. no. K1231). The plasmids were recovered using the PureLink Quick Plasmid Miniprep kit (Life Technologies Corporation, cat. no. K210010) and sequenced.

### Quantitative Reverse Transcription-Polymerase Chain Reaction (qRT-PCR)

To confirm the presence of the transcripts found by RNA-Seq, qRT-PCR was performed using the NCode™ miRNA First-Strand cDNA Synthesis and qRT-PCR kit (Life Technologies Corporation, cat. no. MIRQ-100) according to the manufacturer´s instructions. As starting material we used 1 µg RNA obtained from infection or *in vitro* growth conditions. Briefly, a poly A tail was added to RNA of sizes <200 nt using the poly A polymerase and incubated at 37°C for 15 min. The polyadenylated RNA was used for cDNA synthesis using the SuperScriptIII Reverse Transcriptase, and the reaction was incubated for 50 min at 50°C. Finally, the synthesized cDNA was used for qRT-PCR reactions using the Platinum SYBR Green qPCR SuperMix-UDG with the corresponding oligonucleotides (Table S2). The cycling programme consisted of 50°C for 2 min; 95°C for 2 min; 40 cycles of 95°C for 15 seconds and 65°C for 60 seconds using the Step One Plus^TM^ System (Life Technologies Corporation, cat. no. 4376600). For relative quantification we used the method ΔΔ Ct. The C_T_ values from all qRT-PCR reactions were obtained and analysed in triplicate to confirm the existence of ncRNAs. 5S rRNA was used as endogenous standard to normalize the expression levels of the tested sRNA candidates.

### *In silico* Analysis

The genome of *N. brasiliensis* was analysed to predict regulator ncRNAs using the non-coding RNA characterization (nocoRNAc) software, which incorporates several procedures to detect transcript characteristics. For example, for the detection of the transcript´s 3’-end, nocoRNAc uses the TransTermHP program, which predicts Rho independent end signals. In addition, transcript´s 5’-end are predicted by the detection of destabilized regions in the genomic DNA [15].

## RESULTS

### Induction of Murine Actinomycetoma

To induce infection, 10 male CD-1 mice (eight weeks of age, 25 g) were inoculated in the plantar pad with a *N. brasiliensis* suspension as described in Materials and Methods. Two months after bacterial inoculation, mice exhibited typical signs of actinomycetoma Fig. (**1A**) and were sacrificed. Under sterile conditions and by a skin-deep incision with a scalpel, the purulent material containing numerous grains (consisting of bacterial cumulus) was recovered. The presence of grains was verified by light microscopy Fig. (**1B**). sRNAs were run in a denaturing 12% poly-acrylamide gel to verify its quality (Fig. S1). The absorbance ratios are showed in (Table **1**).

### Global Non-coding RNA Profiles in *N. brasiliensis*

To detect the expression of ncRNAs in *N. brasiliensis* the transcriptome of < 200 nt isolated from actynomycetoma and from *N. brasiliensis* grown on BHI agar for eight days, was subjected to next-generation sequencing on an Illumina platform for 36 bp single-end reads. An outline of the procedures used in this study is presented in Fig. S1. The sequences showing no hits in the mouse genome (32,062,154) were subjected to a BLAST (basic local alignment search tool) search against the *N. brasiliensis* genome resulting in 11,024,619 reads. A Student T-test between reads obtained from actinomycetoma and *N. brasiliensis* grown *in vitro* was done for the in-depth analysis (p < 0.05 values) to determine non-coding RNA candidates. Under this statistical criterion, 70 sequences present in both conditions were identified as potential ncRNAs because they were localized in IGRs or corresponded to antisense of *N. brasiliensis* genes. Table (**2**) presents the localization of 18 of these sequences that were mapped in the *N. brasiliensis* genome and were chosen due to their differential expression in both conditions. Twelve out of these 18 transcripts are encoded in IGRs, three transcripts corresponded to tRNA-derived fragments, and one was overlapped between an IGR and the 5´end 23S rRNA. Finally, two transcripts mapped in the antisense direction of two genes, one of which corresponded to a putative non-ribosomal peptide synthetase and the other to an OmpR family two-component response regulator.

A searching of ORFs by the ORF finder program (NCBI) was performed in order to confirm these small RNAs are not encoding small proteins. After this analysis we found only Nbnc-8 encoding a possible small peptide having 11 amino acids, but it did not show any known protein similarity and its nucleotide sequence is only present in *N. brasiliensis* and *N. seriolae*.

### The Expression of Most ncRNAs was Increased *in vivo* Compared with *in Vitro*

Table (**3**) presents the differential expression values under *in vivo* and *in vitro* conditions of 10 transcripts selected from Table (**2**). These transcripts were selected based on the highest fold changes between two conditions. The differential expression change of these RNAs was confirmed by qRT-PCR Fig. (**2**). The size of these transcripts was determined and RACE, as shown in Table (**3**). The transcripts Nb-tRFthr, Nb-tRFmet, Nbnc-3, Nbnc-5, Nb-tRFcys, Nbnc-10 Nb-rRF_23S_ exhibited increased expression *in vivo* compared with *in vitro*. The remaining ncRNAs (Nbnc-4, Nbnc-7 and Nbnc-8) exhibited an increased expression *in vitro* compared with *in vivo* conditions. Nbnc-3 and Nbnc-5 were upregulated *in vivo* by RNA-Seq; however results were inconsistent by qPCR.

### tRNA-Derived Fragments were Highly Expressed *in Vivo*

In contrast to tRNA derived fragments in other organisms including archeae and humans that are derived from the 5´end, the *Nocardia* tRNA fragments belonging to threonine, methionine and cysteine tRNAs (Nb-tRFmet, Nb-tRFcys, and Nb-tRFthr) identified here are derived from the 3´ end of the tRNAs Fig. (**3**) [17]. After the analysis of diverse Nb-tRFthr clones we found that this transcript sequence corresponds to a fragment overlapping the *N. brasiliensis* tRNA^Thr^ and it ends 10 nt downstream (coordinates 8,659,485-8,659,534 in *N. brasiliensis* genome).

### A Fragment Derived from 23S rRNA (Nb-rRF_23S_) is Distributed in Different Actinomycetes

Among the transcripts that were mainly expressed under *in vivo* conditions, we found Nb-rRF_23S_. Surprisingly, this fragment was located within three of the nine copies of the *N. brasiliensis* rRNA operon overlapping a part of an IGR and the 5´ end of the 23S rRNA gene Fig. (**4**). In contrast to most of ncRNAs detected that range in size from 50 to 300 nt, the transcript size was 466 nt, as determined by RACE experiments.

To investigate the phylogenetic distribution of this transcript in different actinomycetes, the BLAST algorithm was used. We identified the presence of a corresponding sequence in pathogenic and non-pathogenic bacteria belonging to the Actinomycetales order, including the *Nocardia, Mycobacterium, Rhodococcus* and *Tsukamurella* genera. The most conserved region obtained in the alignment was used to construct a phylogenetic tree, which is shown in Fig. (**5**). With the exception of *Rhodococcus equi* 103S, a tendency to form independent clades corresponding to each genus was observed. In several *Nocardia* and other actinomycetes species, the presence of a corresponding sequence has been observed in more than one copy of the rRNA operon.

### Prediction of ncRNAs Using Bioinformatic Tools

The secondary structure of *N. brasiliensis* ncRNAs was predicted using the mfold programme [16]. Results of this application are showed in Figs. S3-S11.

As an alternative approach to searching for ncRNAs in *N. brasiliensis*, an *in silico* analysis was performed using the nocoRNAc programme [15]. According to this software, *N. brasiliensis* contains 146 ncRNAs genes (Table S1), 106 of which are encoded in IGRs and 40 are antisense-localized RNAs of genes with putative function, including three lipases and three oxidoreductases likely involved in virulence.

Interestingly, by comparing the ncRNAs predicted by the nocoRNAc programme and those found by RNA-Seq analysis, four sequences (4 of the 70 RNAs differentially expressed) were found in common Fig. (**6**), and these are localized to IGRs. The expression of three of these ncRNAs was increased in the *in vitro* conditions (including Nbnc-4), whereas one ncRNA was expressed at similar levels in both conditions. The low number of ncRNAs shared by both methods could be due to potential false-positive predictions of the nocoRNAc programme. Moreover, the expression of many other ncRNAs is probably dependent on other experimental conditions, which were not the purpose of this work.

Ten potential *N. brasiliensis* ncRNAs (showed in Table **3**) of known sizes were subjected to the IntaRNA programme in order to predict the corresponding mRNAs target [18]. This programme enables the prediction of interactions in three regions of the mRNA target: start codon, coding sequence, and stop codon. The data from this analysis are shown in Tables S3-S6. The ncRNA Nbnc-3 (Tables S3A-C) was predicted to interact with the start codon of a Mce-family protein, with the start codon and coding sequence of a mycolyltransferase involved in *Rhodococcus equi*‘s virulence [19], and with the stop codon of a carboxylesterase, a known *M. tuberculosis* virulence factor [20]. Moreover, Nbnc-10 (Tables S4A-B) was predicted to interact with the start codon of a lipase and an epoxide hydrolase, both *P. aeruginosa* virulence factors [21]. Nbnc-10 also interacted with the stop codon of a glycosyl transferase that is essential in *Streptococcus pneumoniae* lung infections [22].

Furthermore, Nb-tRFcys (Tables S5A-C) was predicted to interact with the start codon of several mRNAs related to virulence, including dipeptidyl aminopeptidase [23], sodium hydrogen exchanger [24], TetR family of transcriptional repressors [25], and aldehyde dehydrogenase [26]. In addition, Nb-tRFcys was predicted to interact with the coding sequence of a dipeptidyl aminopeptidase, and with the stop codon of the protocatechuate 34-dioxygenase subunit alpha [27], a lipase [28], and an amidase [29]. Finally, Nb-rRF_23S_ was predicted to interact with the stop codon of a chloramphenicol resistance protein.

## DISCUSSION

The *Nocardia* genus currently contains approximately 86 species, of which approximately half are recognized as human and/or other animal pathogens [30]. *N. brasiliensis* appears to be the most virulent bacterium within the *Nocardia* genus, as suggested by the high number of actinomycetoma cases caused by this species [12, 31, 32]. The chronic evolution of the disease caused by this bacterium first exhibits a asymptomatic infection period followed by a slow bacteria adaptation process to environmental changes within the host that allow this pathogen to survive the adaptive immune response.

Researchers have demonstrated that many organisms possess ncRNAs molecules that function as regulators of gene expression. These molecules have been identified in prokaryotes, eukaryotes and archaea [2]. In bacteria, many of these new ncRNAs have been identified as integral components of the bacterial stress response with well-established roles in bacterial survival within the host. RNA-based regulatory elements that control pathogenesis include riboswitches, which are 5’-untranslated regions of mRNAs and small ncRNAs [33].

In 2009, the first complete experimental confirmation of ncRNAs in mycobacteria was published, revealing five *trans*-encoded and four *cis*-encoded ncRNAs in *Mycobacterium tuberculosis* H37Rv expressed in the exponential and stationary growth phases. Under different growth conditions, *M. tuberculosis* expresses a plethora of non-coding transcripts, most of which play unknown roles. For example, B11 and F6 are encoded in IGRs, and the deletion of B11 and F6 is lethal and leads to reduced *M. tuberculosis* growth, respectively [34].

*Streptomyces coelicolor* is a non-pathogenic actinomycete that represents a bacterial model that was significantly useful in uncovering the regulatory mechanisms of non-coding RNAs. Vockenhuber *et al.* [35] reported approximately 63 ncRNAs in this bacterium, including 29 *cis-*encoded antisense RNAs.

In this study we identified 70 ncRNAs expressed either during experimental actinomycetoma or *in vitro* growth of *N. brasiliensis*, 18 of which showed differential expression between these two conditions. The differential expression of 10 of these transcripts was confirmed by qRT-PCR analysis and its size determined by RACE. Among these, three decreased their expression during infection while seven exhibited increased expression levels during actinomycetoma. The increased expression of these ncRNAs suggests a role in the infection process. While the decreased expression suggests either that they have no role in the infection process or that they have a role by the mean of up-regulating of *N. brasiliensis* virulence factors. The transcripts identified may contribute to an adaptive response of *N. brasiliensis* during the host infection that enables confrontation of stressors due to changes in the availability of nutrients (such as iron), temperature (37ºC), pH (approximately 7.0) and an aggressive immunological system.

Regarding the nature and putative roles of the ncRNAs identified in this study the transcripts encoded within IGRs probably represent *trans-*acting ncRNAs that regulate the expression of mRNAs throughout different regions of the bacterial genome. Concerning Nbnc-8 further studies are necessary in order to confirm it is encoding a small protein as reported by Hemm *et al.* [36]. Most sRNAs found in bacteria are *trans*-acting transcripts. Due to localization and low base-complementary, it is difficult to determine the corresponding messenger targets of ncRNAs and therefore their functions.

The transcripts Nbnc-13 and Nbnc-15 mapped in the antisense direction of genes encoding a putative non-ribosomal peptide synthetase (NRPS) and a response regulator of the OmpR family respectively. The NRPSs are implicated in the synthesis of many secondary metabolites, including siderophores that play important roles in the uptake of environmental iron [37]. Interestingly, siderophores are considered virulence factors. In *M. tuberculosis*, a siderophore export system has been described as essential for virulence [38]. *N. brasiliensis* contains genes coding for NRPS, and Nbnc-13 could participate in the regulation of siderophore production. However, this hypothesis remains to be confirmed.

OmpR activates the expression of external membrane porins, including OmpF, OmpC, OmpS1 and OmpS2 (non-specific porins that regulate the influx of water, glucose and ions into the cell). This regulatory pathway has been described in *Salmonella enterica* and *S. typhimurium* [39]. MicF is an ncRNA that regulates OmpF synthesis in *Escherichia coli* under different stress conditions (e.g., changes in osmolarity and pH) [40]. Considering that Nbnc-15 is localized in an antisense orientation to OmpR we propose that it could regulate the master regulator OmpR. The decreased expression of Nbnc-15 during infection could induce an increase in OmpR expression and accordingly induce porin synthesis providing an advantage by increasing nutrient entry into bacterial cells.

The three tRNA-derived fragments belonging to tRNA^Cys^, tRNA^Thr^ and tRNA^Met^, identified in this study were mainly expressed during experimental actinomycetoma. The three fragments correspond to the 3´ends of the transcripts and probably represent the processing products of the tRNAs. The Nb-tRFthr transcript sequence overlapping the tRNA^Thr^ found in this study could correspond to a precursor tRNA fragment after its processing as it ended 10 nt downstream. In *Haloferax volcanii*, various tRNAs are processed to produce tRNA-derived fragments when the microorganism is subjected to different types of stress [7]. However, another possibility is the existence of a posttranscriptional modification and stable structure that interfere with cDNA synthesis as described other authors. Further studies are needed to verify the true nature of these fragments [41, 42].

Finally, we identified a 466 nt transcript (Nb-rRF_23S_) overlapping an IGR and the 5´ end of the 23S rRNA that was up-regulated in actinomycetoma compared to *in vitro* conditions. This molecule is likely a product of RNA processing that results in a transcript with a putative regulatory function during infection. To date, we are unaware of any reports of regulatory ribosomal RNAs. Interestingly, this transcript is only found in three of the nine ribosomal operons in *N. brasiliensis*; whether these three transcripts are functional is still unknown. Surprisingly, this sequence is highly conserved in other pathogenic bacteria (*Rhodococcus*, *Tsukamurella* and *Mycobacterium*) as shown in Fig. (**5**). This finding suggests that 23S rRNA may be processed to yield a fragment with regulatory activity that could be involved in the pathogenesis of some actinomycetes.

This work represents the first inquiry of the presence of ncRNAs in *N. brasiliensis*, an actinomycete that commonly causes human subcutaneous and lung infections. One of the experimental conditions explored was the murine actinomycetoma, which closely simulates the bacterial stress conditions when *N. brasiliensis* infects a human host.

## CONCLUSION

The study of the differential expression of ncRNAs during *in vitro* culture and *in vivo* infection allowed us the identification of ncRNAs and represent a valuable strategy to start our understanding of *N. brasiliensis* pathogenesis and the establishment and evolution of actinomycetoma. The function of these transcripts and their mechanisms of action in the pathogenesis process of this bacterium remain to be determined. In the future this kind of studies will provide information useful when proposing new therapeutic approaches to combat actinomycetoma and/or nocardiosis.

## Figures and Tables

**Fig. (1):**
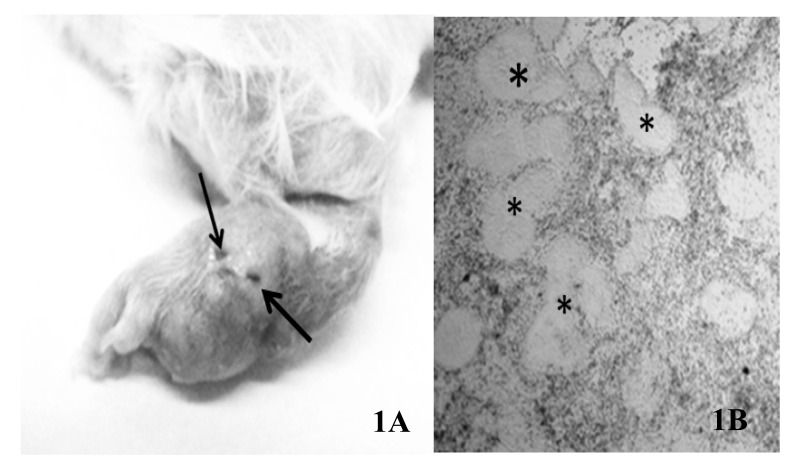
A) Experimental actinomycetoma in a murine model after eight weeks of evolution, showing deformation, swollen lesions and sinus tracts (arrows) in the cutaneous and subcutaneous tissues. B) Microscopic preparation of purulent material recovered from mice infected with *N. brasiliensis* contrasted with lugol, showing several grains (asterisks) (magnification 40X).

**Fig. (2):**
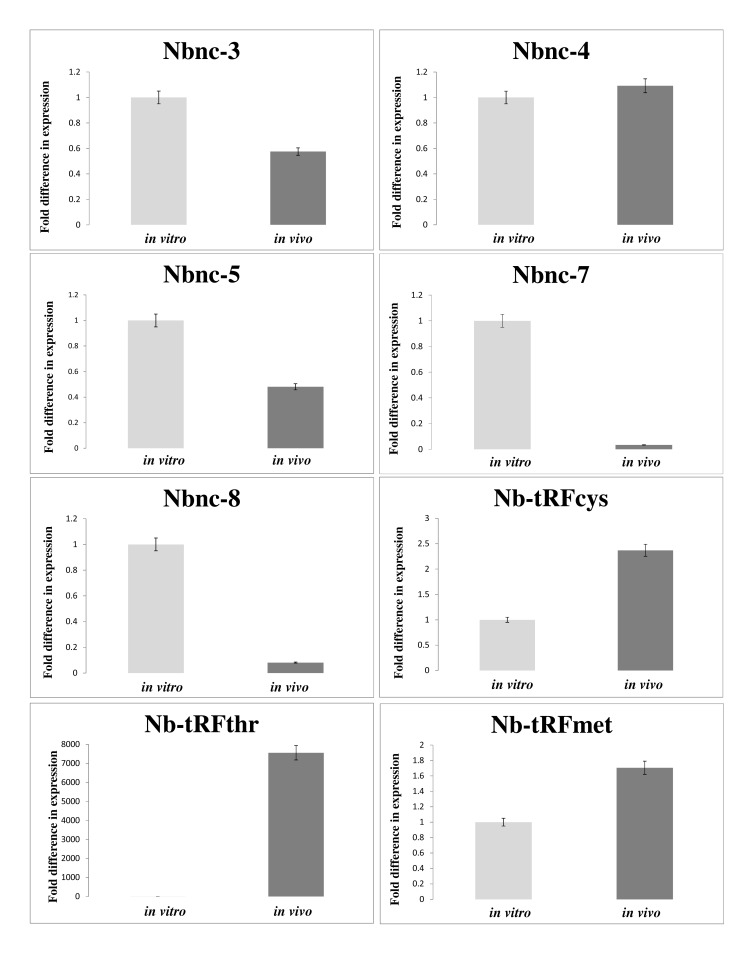
The validation of the RNA-Seq results by qRT-PCR and changes in the *in vitro* and *in vivo* expression levels. Differential expression levels of eight selected transcripts were determined. The Y-axis values indicate the fold change differences in expression and were calculated using the 2^ΔΔCt^ parameter. qRT-PCR data represent the mean of three independent experiments. Error bars represent the standard deviation.

**Fig. (3):**
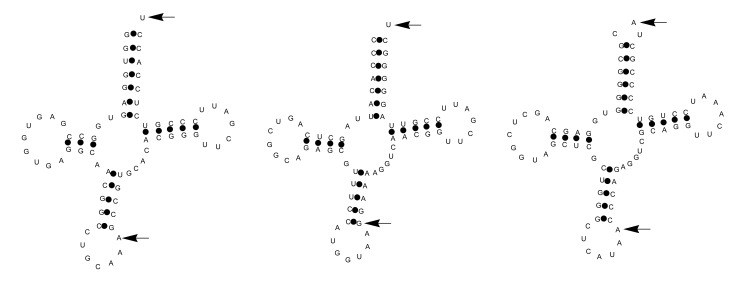
The secondary structure of tRFs expressed in *N. brasiliensis* and obtained by *in silico* analysis [17]. From left to right: Nb-tRFcys, Nb-tRFmet and Nb-tRFthr. Arrows indicate the processing points on the tRNAs used to produce the fragments with probable regulatory activity.

**Fig. (4):**
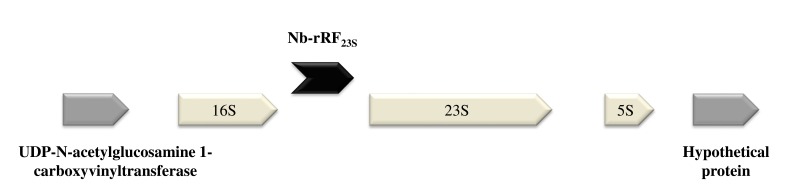
The location of Nb-rRF_23S_ within the *N. brasiliensis* genome. This transcript was located within three of the nine copies of the *N. brasiliensis* ribosomal operon.

**Fig. (5):**
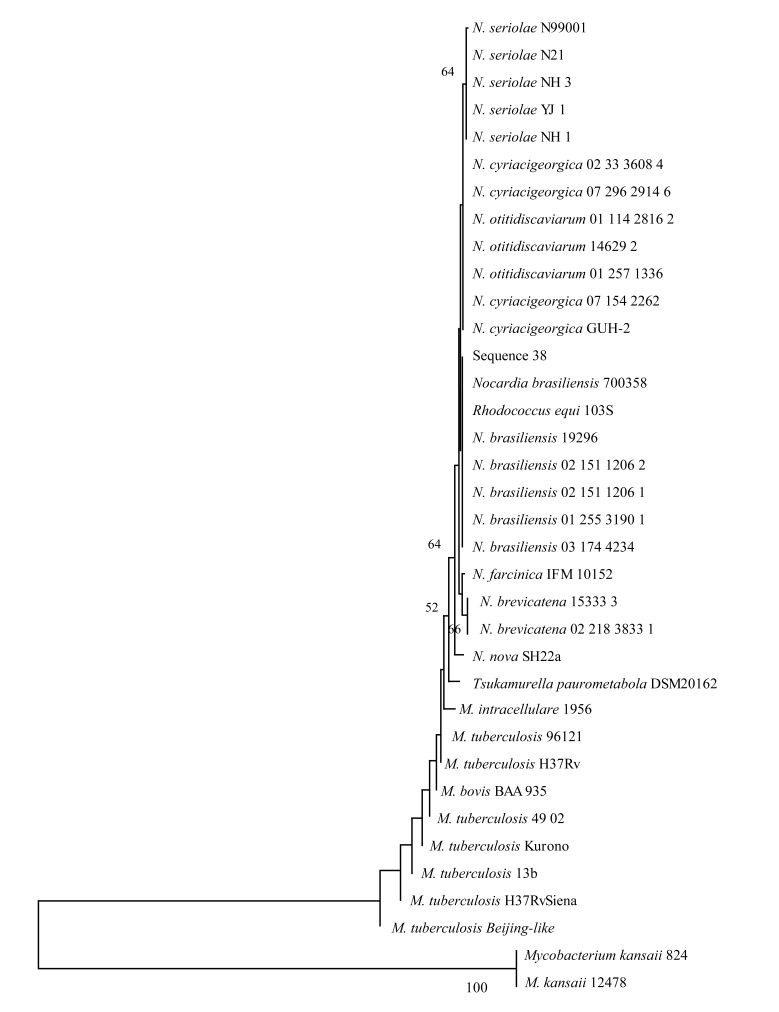
A dendrogram of actinomycetes that share the Nb-rRF_23S_ sequence. The dendrogram was obtained by comparing the sequences of Nb-rRF_23S_ among diverse pathogenic actinomycetes, including *M. tuberculosis* H37Rv. A highly conserved 91-nt region was used to construct this tree.

**Fig. (6):**
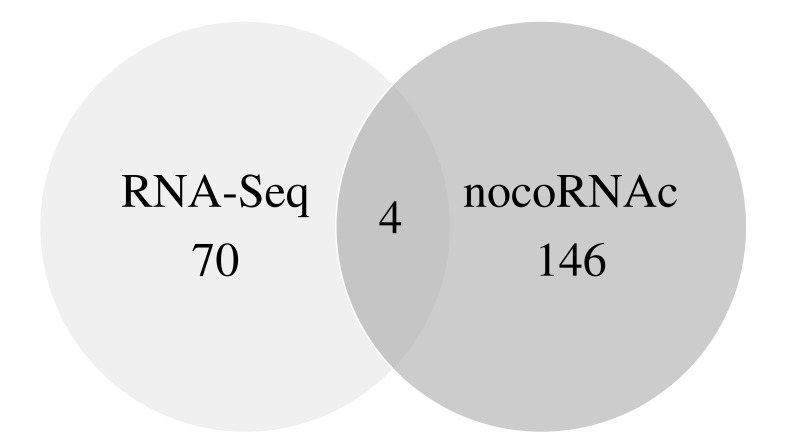
Comparison of *N. brasiliensis* ncRNAs identified by RNA-Seq vs *in silico* analysis (15). The diagram compares the results for the *N. brasiliensis* chromosome. Four sRNA genes were identified using both methods.

**Table 1:** Quantitation and absorbance ratios of sRNA used to RNA-Seq analysis. Samples 1-6 correspond to sRNAs obtained from *N. brasiliensis* grown *in vitro*. Samples 7-10 correspond to sRNAs obtained from actinomycetoma.

Samples	Concentration (ng/ul)	Absorbance ratios 280/260
1	233.4	1.98
2	223.52	2.02
3	90.44	1.87
4	207.12	1.97
5	230.19	1.98
6	148.89	1.99
7	470.53	1.88
8	509.18	1.91
9	1192.74	1.92
10	634.67	1.98

**Table 2:** Localization of differentially expressed transcripts in the *N. brasiliensis* genome *in vivo* and *in vitro*, as identified by RNA-Seq. Most sequences were localized in intergenic regions (two were found in antisense gene regions, three corresponded to tRFs and one was overlapped between an IGR and the 5´ end of the 23S rRNA).

ncRNA (RNA-Seq number)	Assigned number in this work	Position in genome	Intergenic region (IGR)	Antisense
PC-5p-1227_2433	Nbnc-3	431121- 431156	Putative hydrolase < Nbnc-3 > hypothetical protein	
PC-3p-38_178098	Nbnc-4	2052731- 2052756	FAD-dependent pyridine nucleotide-disulphide oxidoreductase < Nbnc-4 > valyl-tRNA ligase	
PC-5p-7930_296	Nbnc-5	7645558- 7645594	Hypothetical protein < Nbnc-5 > hypothetical protein	
PC-3p-79_77742	Nbnc-7	1392314- 1392294	Hypothetical protein < Nbnc-7 > hypothetical protein	
PC-5p-206_24405	Nbnc-8	2467033- 2467051	Hypothetical protein < Nbnc-8 > pyruvate dehydrogenase subunit E1	
PC-3p-27015_78	Nbnc-10	2510229- 2510251	Putative hydrolase <Nbnc-10 > hypothetical protein	
PC-3p-1044_3001	Nbnc-11	8615680- 8615702	Ferredoxin reductase < Nbnc-11 > putative phosphohistidine phosphatase	
PC-5p-1215_2462	Nbnc-12	9097543- 9097565	Hypothetical protein < Nbnc-12 > 6-phosphogluconate dehydrogenase-like protein	
PC-5p-36673_57	Nbnc-13	6046137- 6046162		Putative non-ribosomal peptide synthetase
PC-3p-12562_178	Nbnc-14	7645737- 7645773	Hypothetical protein < Nbnc-14 > hypothetical protein	
PC-5p-4197_599	Nbnc-15	2139099- 2139080		OmpR family two-component response regulator
PC-5p-5913_410	Nbnc-17	8421241- 8421211	Hypothetical protein < Nbnc-17 > hypothetical protein	
PC-3p-733_4626	Nbnc-18	2646061- 2646093	Cell division protein MraZ < Nbnc-18 > 16S rRNA m(4)C1402 methyltransferase	
PC-3p-238_20255	Nb-tRFthr	8659529- 8659494		^a^tRNA^thr^
PC-5p-8894_263	Nb-tRFmet	1323299- 1323263		^a^tRNA^met^
PC-5p-26921_79	Nb-tRFcys	6642901- 6642936		^a^tRNA^cys^
PC-3p-6945_343	Nb-rRF23S	1449884- 1449906	rRNA-16S ribosomal RNA < Nb-rRF23S > rRNA-5S ribosomal RNA	23S ribosomal

^a^These sequences represent tRNA-derived fragments.

**Table 3:** Characteristics of the ncRNAs differentially expressed by *N.brasiliensis in vivo* and *in vitro*. The size and localization of 10 transcripts within the *N. brasiliensis* genome were determined using RACE.

Assigned number in this work	Reads determined by RNA-Seq *in vivo*	Reads determined by RNA-Seq *in vitro*	Log2	Fold changes	Position in the genome, determined by RACE (nt)	Size determined by RACE (nt)
Nb-tRFthr	18,206	2,058	-3.14	8.8	8659494-8659529	36
Nb-tRFmet	1,784	16	-6.8	111.4	1323299-1323263	36
Nbnc-3	5,929	230	-4.68	25.6	431089-431187	98
Nbnc-4	6,117	26,839	2.13	4.3	2052706-2052756	51
Nbnc-5	2,651	17	-7.28	155.4	7645546-7645594	49
Nb-tRFcys	630	11	-5.75	53.8	6642901-6642936	36
Nbnc-7	619	5,321	3.1	8.5	1392271-1392314	44
Nbnc-8	241	2,204	3.19	9.1	2467007-2467083	77
Nb-rRF23S	691	31	-4.47	22.1	1449866-1450332	466
Nbnc-10	502	9	-5.64	49.8	2510219-2510295	76

## LIST OF ABBREVIATIONS

BLAST: = Basic local alignment search tool
Cys: = Cysteine
IGR: = Intergenic region
Met: = Methionine
Nb-rRF_23S_: = *Nocardia brasiliensis* 23S ribosomal RNA-fragment
Nb-tRF: = *Nocardia brasiliensis* transfer RNA-fragment
Nbnc: = *Nocardia brasiliensis* non-coding RNA
ncRNA: = non-coding RNA
nocoRNAc: = non-coding RNA characterization
nt: = Nucleotides
qRT-PCR: = Quantitative reverse transcription polymerase chain reaction
RACE: = Rapid amplification of cDNA ends
RNA-Seq: = RNA sequencing
rRNA: = ribosomal RNA
sRNA: = small RNA
Thr: = Threonine
tRNA: = transfer RNA
